# Amino-Terminated Poly(propylene oxide) as an Interfacial Dispersant for Low-Conductivity Silica/Carbon Black Hybrid-Filled Natural Rubber Composites

**DOI:** 10.3390/polym17081023

**Published:** 2025-04-10

**Authors:** Jiahui Mei, Ying Liu, Youliang Zhou, Depeng Gong, Lili Wu, Chaocan Zhang

**Affiliations:** School of Materials Science and Engineering, Wuhan University of Technology, Wuhan 430070, China; m2bd4l@163.com (J.M.); ly2860495025@163.com (Y.L.); zyl_2013@whut.edu.cn (Y.Z.); gdp@whut.edu.cn (D.G.); polym_wl@whut.edu.cn (L.W.)

**Keywords:** natural rubber, carbon black, silica, interfacial dispersant, electrical resistivity

## Abstract

Natural rubber is widely used in various engineering fields due to its excellent properties, particularly as an anti-corrosion and wear-resistant lining for metal pipelines. The defects in rubber linings are typically detected using the electrical spark test. Carbon black can enhance the strength, modulus, and wear resistance of natural rubber. However, conventional carbon black-filled natural rubber composites exhibit a certain level of electrical conductivity, making them unsuitable for defect detection via the electrical spark test. In this study, a silica/carbon black hybrid filler system was selected, and different types of amino-terminated poly(propylene oxide) were employed as novel interfacial dispersants to develop a low-conductivity natural rubber composite suitable for electrical spark testing while meeting general industrial mechanical performance requirements. The role of amino-terminated poly(propylene oxide) was first explored in a pure carbon black system, and then the optimized types and dosages of amino-terminated poly(propylene oxide) were added into a mixed filler system of silica and carbon black to explore the silica dosage that could balance the resistivity and mechanical properties. The results showed that the amino-terminated poly(propylene oxide) could improve the dispersion of carbon black and silica, thus increasing the mechanical properties of natural rubber composites. In the pure carbon black system, the tensile strength of natural rubber composites increased by 18.2%, the 300% modulus increased by 74.6%, and the Akron abrasion decreased by 42.7%. In the mixed filler system, the tensile strength of the natural rubber composites with 20 phr of silica and 30 phr of carbon black was 24.03 MPa, the 300% modulus was 15.16 MPa, and the Akron abrasion was 0.223 cm^3^. In addition, the volume resistivity was 5.52 × 10^9^ Ω·cm, which is suitable for detecting defects with the spark test.

## 1. Introduction

Natural rubber (NR) is widely used in various applications, such as tire treads and anti-corrosion wear-resistant layers, due to its excellent resilience, plasticity, and electrical insulation properties. However, the mechanical properties of natural rubber are no longer sufficient to support its application in many engineering fields. Therefore, the incorporation of fillers to enhance the performance of natural rubber is of great importance [1,2,3,4]. Among various rubber fillers, carbon black (CB) is the most widely used due to its excellent reinforcing capability and cost-effectiveness. It enhances the strength, modulus, and wear resistance of natural rubber [5,6,7]. In addition, silica (SiO_2_) is an excellent non-black filler that is commonly used in tire tread production, as it can reduce the rolling resistance of rubber composites and improve wet skid resistance [8,9,10]. Natural rubber composites are commonly used as protective coatings for metal materials, providing anti-corrosion and wear-resistant properties. However, the presence of defects such as pores and voids can significantly compromise their protective performance [11,12]. High-frequency electrical spark testing is typically employed as an effective method for detecting defects in rubber composites (refer to BS 6374-5, ASTM D5162, and GB/T 18241). However, carbon black with a small particle size and high reinforcement efficiency generally exhibits strong electrical conductivity [13,14,15]. As a result, carbon black/natural rubber composites tend to undergo self-discharge, making them unsuitable for defect detection using electrical spark testing. There are very few reports on the relationship between electrical spark testing and resistivity [16]. Introducing insulating fillers to increase the resistivity of rubber composites is a potential approach. Thaptong et al. [17] investigated the effects of different SiO_2_ contents on the electrical properties and other performance aspects of CB/SiO_2_ hybrid-filled tread compounds. The results showed that when the SiO_2_ content was 48 phr, the surface resistivity reached its highest value of 3.7 × 10^11^ Ω, whereas at 24 phr SiO_2_, the surface resistivity was at its lowest, measuring 6.9 × 10^5^ Ω. The corresponding tensile strength was only 20.8 MPa, and the elongation at break was 392%. Wang, L.’s team [18] prepared SiO_2_/CB-filled solution-polymerized styrene–butadiene rubber (S-SBR) composites using a reactive blending method and investigated their mechanical and anti-static properties. When the SiO_2_ content was increased from 0 to 35 phr, the surface resistivity and volume resistivity of the composites increased from 10^7^ to 10^9^ Ω. As the SiO_2_ content was further increased from 35 phr to 70 phr, the surface resistivity and volume resistivity increased from 10^9^ to 10^15^ Ω. When both carbon black and silica were present at 35 phr, the composite exhibited a tensile strength of 22.2 MPa and an elongation at break of 409%. Al-Ghamdi et al. [19] investigated the effect of SiO_2_ content on certain properties of CB/SiO_2_ hybrid filler-reinforced natural rubber composites. The introduction of the silica phase and its increasing content led to an increase in both volume resistivity and surface resistivity. When the SiO_2_ content was increased from 3% to 7% of the total filler amount, the volume resistivity increased from 3.75 × 10^12^ Ω·m to 6.45 × 10^14^ Ω·m. This indicates that the incorporation of silica can effectively enhance the volume and surface resistivity of rubber composites, and the silica content is a crucial factor. However, none of the above studies have addressed spark testing.

In this paper, a CB/SiO_2_ hybrid filler system was used to improve the electrical resistivity of rubber composites aiming to prepare natural rubber composites suitable for spark detection, and due to the large number of polar groups on the surface of silica and large cohesive energy, it is difficult to disperse in non-polar natural rubber [20]. We used a new class of interfacial dispersants—amino-terminated poly(propylene oxide)—to simultaneously improve the carbon black and silica dispersibility of carbon black and silica at the same time to increase the properties of natural rubber composites. Firstly, three kinds of amino-terminated poly(propylene oxide) with different molecular weights of mono- and diamino-amino groups were added to the pure carbon black system, and the effects of their types and dosages on the filler dispersibility, vulcanization properties, mechanical properties, and other properties of the carbon black/natural rubber composites were investigated. Subsequently, the optimized types and amounts of amino-terminated poly(propylene oxide) were added into the CB/SiO_2_ hybrid filler system to study the effects of different silica and carbon black ratios on the properties of the natural rubber composites, and a suitable silica content was investigated to balance the mechanical properties and resistivity of the natural rubber composites.

## 2. Materials and Methods

### 2.1. Experimental Materials

Natural rubber (SCR-WF, first grade standard rubber, all latex, impurity content of 5%) was supplied by Yunnan Natural Rubber Industry Group (Sipsongpanna, China). Carbon black (N220) was provided by Cabot Corporation (Shanghai, China). Amino-terminated poly(propylene oxide) was manufactured by Huntsman Corporation (Shanghai, China), with three different grades: M2005 (monoamino-terminated, molecular weight ~2000), D400 (diamino-terminated, molecular weight ~400), and D2000 (diamino-terminated, molecular weight ~2000). Precipitated silica (VN3 GR) with an average particle size of 20–30 nm and a BET surface area of 175 m^2^/g was purchased from Evonik Industries (Essen, Germany). Bis-[γ-(triethoxysilyl)propyl] tetrasulfide (Si-69) was sourced from Dongguan Kangjin New Materials Co., Ltd. (Dongguan, China). Alkyl sulfonic phenyl ester (T-50) was supplied by Zhengzhou Yufeng Nanomaterials Co., Ltd. (Zhengzhou, China). Other rubber additives, including zinc oxide (ZnO), stearic acid (SA), N-(1,3-dimethylbutyl)-p-phenylenediamine (4010NA), 2,2,4-trimethyl-1,2-dihydroquinoline polymer (RD), N-tert-butyl-2-benzothiazolesulfenamide (NS), and sulfur (S), were all industrial-grade.

### 2.2. Preparation of Natural Rubber Composites

The compounding process was carried out in a torque rheometer at a rotor speed of 50 r/min. First, the temperature was set to 80 °C, and NR was masticated for 5 min. Then, the temperature was increased to 110 °C, followed by the addition of carbon black N220 and amino-terminated poly(propylene oxide) and mixed for 10 min. Subsequently, stearic acid, zinc oxide, and antioxidants RD and 4010NA were sequentially added and mixed. Finally, the plasticizer T50 was introduced and mixed for 5 min before discharging the compound. Sulfur and accelerator NS were mixed on an open mill, left to stand for 12 h, and then molded. The molding process was conducted at 145 °C for the optimal vulcanization time determined experimentally. For the carbon black/silica hybrid filler system, NR was first masticated at 80 °C for 5 min, and then the temperature was increased to 150 °C, followed by the addition of silica and Si-69 and mixed for 10 min. Subsequently, carbon black and amino-terminated poly(propylene oxide) were introduced and mixed. The remaining steps were the same as described above. The formulations of all composite materials are summarized in Table 1.

### 2.3. Characterization

The tensile test of the composites was conducted at 25 °C using a universal testing machine (MTSDW-20, MTS Testing Instruments Co., Ltd., Jinan, China) in accordance with GB/T 528-2009, with a tensile rate of 500 mm/min.

The hardness was measured using a Shore A durometer (LX-A, Shanghai Gaozhi Precision Instrument Co., Ltd., Shanghai, China) according to GB/T 531.1-2008.

A rubber process analyzer (RPA) (RPA-8000, Taiwan GaoTie Co., Ltd., Taiwan, China) was used to perform strain sweep tests on the uncured NR composites under conditions of 60 °C and 1 Hz. The strain sweep range was set from 0.1% to 400%.

A dynamic mechanical analyzer (DMA) (TA Q800, TA Instruments, New Castle, USA) was used to evaluate the loss factor (tan δ) of the vulcanized rubber composites. Temperature sweep tests were conducted on rectangular specimens (10 mm × 5 mm × 2 mm) at a frequency of 10 Hz and an amplitude of 40 μm, within a temperature range of −80 to 80 °C, at a heating rate of 3 °C/min.

The curing characteristics of NR/CB composites were measured using a rotorless rheometer (M-3000A, Taiwan GaoTie Co., Ltd., Taiwan, China) in accordance with GB/T 16584-1996. The torque–time curve was recorded at 145 °C.

The surface morphology and filler distribution of the tensile fracture cross-sections of the composites were observed using a scanning electron microscope (SEM) (TESCAN MIRA LMS, TESCAN Trading Co., Ltd., Brno, The Czech Republic) with an accelerating voltage of 20 kV.

The abrasion resistance of the composites was tested using an Akron abrasion tester (CZ-3005, Yangzhou Changzhe Testing Machinery Co., Ltd., Yangzhou, China) according to GB/T 1689-2014. The density (ρ) of the samples was measured according to GB/T 533-2008.

The volume resistivity and surface resistivity were tested using a surface and volume resistivity tester (FT-305, Ningbo Ruikeweiye Instrument Co., Ltd., Ningbo, China) according to GB/T 40719-2021. The test samples were 2 mm thick vulcanized rubber specimens.

Electrostatic spark testing was conducted on vulcanized rubber specimens (2 mm thickness) using a spark tester (ARP-S7, Quzhou Aipu Metrology Instrument Co., Ltd., Quzhou, China) according to GB/T 18241. The applied voltage was set at 6000 V (3000 V/mm). During testing, composites with low resistivity that are unsuitable for spark detection caused immediate spark discharge due to self-discharge. In contrast, for composites suitable for spark detection, sparks were generated only when the air within defects was broken down.

## 3. Results and Discussion

To successfully prepare rubber composites suitable for spark testing while maintaining excellent mechanical properties, carbon black and silica were used as a hybrid filler system. However, the strong polarity of silica makes it incompatible with non-polar natural rubber. To address this issue, amino-terminated poly(propylene oxide) with different molecular weights, including monoamino and diamino types, was incorporated to improve the dispersion of both carbon black and silica.

### 3.1. Effect of Amino-Terminated Poly(propylene oxide) on Carbon Black/Natural Rubber Composites

Carbon black exhibits excellent reinforcing properties for natural rubber, making its dispersion critically important. Initially, we investigated the effects of different types and contents of amino-polyepoxide on the properties of carbon black/natural rubber composites in a pure carbon black system.

#### 3.1.1. Influence of Amino-Terminated Poly(propylene oxide) on the Dispersion of Carbon Black in Natural Rubber

**Figure 1:**
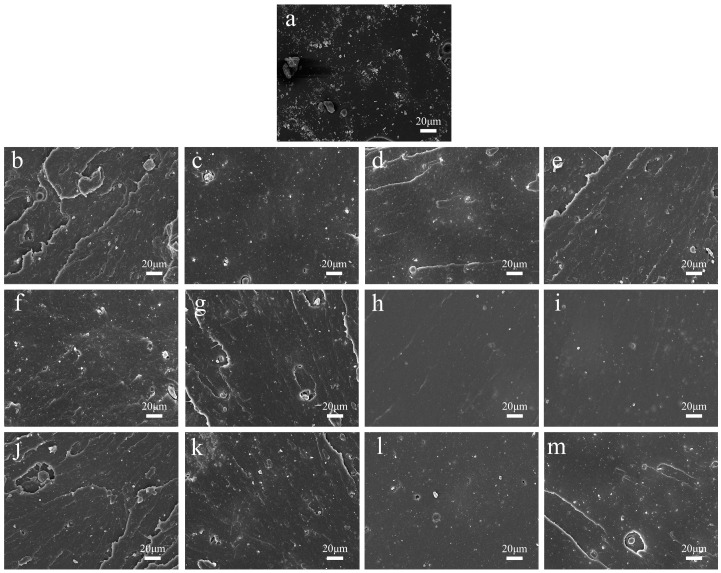
(**a**) SEM image of the blank sample. (**b**–**e**) SEM images of composites with M2005 content ranging from 1 phr to 4 phr. (**f**–**i**) SEM images of composites with D400 content ranging from 1 phr to 4 phr. (**j**–**m**) SEM images of composites with D2000 content ranging from 1 phr to 4 phr.

We prepared carbon black/natural rubber composites with M2005 (monoamino-terminated, molecular weight ~2000), D400 (diamine-terminated, molecular weight ~400), and D2000 (diamine-terminated, molecular weight ~2000) at concentrations ranging from 1 phr to 4 phr. The tensile fracture cross-sections were observed using SEM, and the obtained images are presented in Figure 1. It can be observed that as the content of the three types of amino-terminated poly(propylene oxide) increases from 1 phr to 3 phr, the agglomeration of carbon black gradually weakens. In the images of the blank sample and the samples with 1 phr and 2 phr of amino-terminated poly(propylene oxide), agglomerated CB particles are unevenly distributed on the fracture surface, indicating poor carbon black dispersion. In the images of the samples with 3 phr of amino-terminated poly(propylene oxide), carbon black particles are observed to be uniformly dispersed in the natural rubber matrix. This may be attributed to the formation of hydrogen bonds between the amino groups of amino-terminated poly(propylene oxide) and the carboxyl groups on the surface of carbon black particles. Additionally, the poly(propylene oxide) structure of amino-terminated poly(propylene oxide) is lipophilic, which enhances the compatibility between carbon black and natural rubber, together with the interfacial interactions between them [21,22]. It can also be observed that the fracture surfaces of both the blank samples and the composites with less amino-terminated poly(propylene oxide) content show a large number of rubber matrix tear marks, whereas the fracture surfaces of the composites with an increase in amino-terminated poly(propylene oxide) content up to 3 phr are smooth and the rubber matrix tear marks are significantly reduced, which may be attributed to the fact that amino-terminated poly(propylene oxide) increases the interfacial interactions between the carbon black and the natural rubber. The sample with 4 phr of amino-terminated poly(propylene oxide) exhibits less uniform dispersion compared to the sample with 3 phr. This may be due to excessive amino-terminated poly (propylene oxide), which adversely affects the mixing process of the rubber compound [23]. Additionally, it can be observed that when the content of amino-terminated poly(propylene oxide) is 3 phr, the dispersion of carbon black in the D400 sample is better than that in the M2005 and D2000 samples. This may be because M2005 is mono-amine terminated, whereas D400 is di-amine terminated, resulting in weaker interfacial interactions in the M2005 sample compared to D400. However, despite also being di-amine terminated, D2000 is less effective than D400 in improving carbon black dispersion. This could be due to the larger molecular weight of D2000, which leads to partial compatibility with natural rubber, reducing its effectiveness in enhancing dispersion.

**Figure 2:**
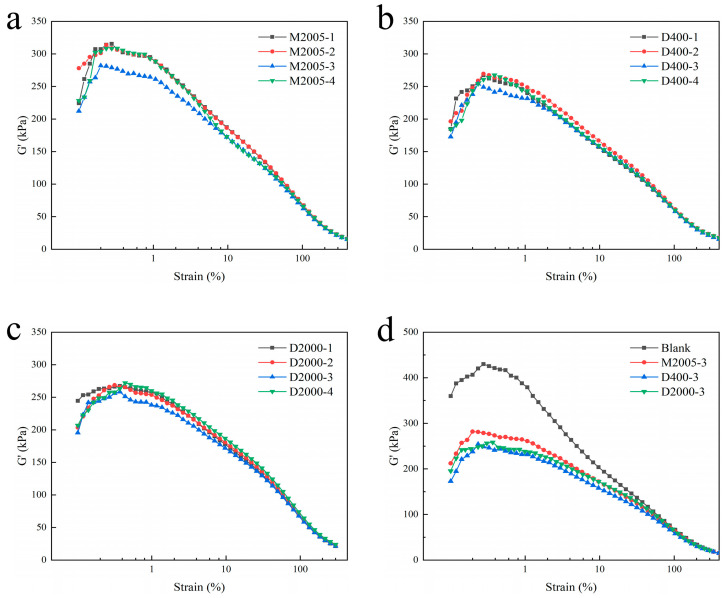
(**a**–**c**) Storage modulus (G′)–strain curves of composites with M2005, D400, and D2000 at loadings of 1–4 phr; (**d**) presents the storage modulus (G′)–strain curves of composites containing 3 phr of the three types of amino-terminated poly(propylene oxide).

The storage modulus (G′) of rubber composites can indirectly reflect the dispersion of fillers. We compounded rubber materials with M2005, D400, and D2000 at loadings ranging from 1 phr to 4 phr. The uncured compounds were tested using a rubber process analyzer (RPA), and the relationship between the shear modulus (G′) and strain of the composites was obtained, as shown in Figure 2. By comparing the difference (ΔG′) between the storage modulus (G′) at small strain and large strain in Figure 2a–c, it was observed that ΔG′ was minimized when the content of all three types of amino-terminated poly(propylene oxide) was 3 phr. The magnitude of ΔG′ reflects the intensity of the “Payne effect” [24]. The Payne effect is caused by the breakdown of the filler network under oscillatory shear and the release of rubber constrained by the filler network. It is commonly used to characterize the dispersion state of fillers [25,26]. Therefore, it can be concluded that the dispersion of carbon black is optimal when the content of the three types of amino-terminated poly(propylene oxide) is 3 phr. From Figure 2d, it can be observed that the ΔG′ values for the composites containing 3 phr of the three types of amino-terminated poly(propylene oxide) follow the order: D400 < D2000 < M2005. This indicates that carbon black dispersion is the most uniform in the D400-based composite, followed by D2000, and the least uniform in M2005. This trend is consistent with the carbon black dispersion observed in the SEM images.

#### 3.1.2. Influence of Amino-Terminated Poly(propylene oxide) on the Vulcanization of Carbon Black/Natural Rubber Composites

A non-rotor rheometer was used to vulcanize the compounded rubber containing three types of amino-terminated poly(propylene oxide) at 145 °C, with contents ranging from 1 phr to 4 phr. The torque–time curves were recorded, and the obtained vulcanization parameters are summarized in Table 2. As the content of the three types of amino-terminated poly(propylene oxide) increased from 1 phr to 4 phr, the scorch time (T_10_) and optimum cure time (T_90_) of the carbon black/natural rubber composites exhibited a decreasing trend. This phenomenon is primarily attributed to the amino groups in amino-terminated poly(propylene oxide), which can act as vulcanization accelerators [27], thereby expediting the entire vulcanization process. Additionally, it can be observed that the T_10_ and T_90_ values of the D400 composite decreased the most, followed by D2000, and lastly M2005. Since D400 and D2000 contain one more amino group than M2005, they exhibit a stronger accelerating effect. However, due to the higher molecular weight of D2000, part of it is compatible with natural rubber and does not contribute to the vulcanization process, resulting in a weaker promoting effect compared to D400. The M_L_ of the carbon black-filled composites increased compared to the blank sample because the addition of amino-terminated poly(propylene oxide) slightly increased the viscosity of the unsulfured compound. Conversely, the M_L_ of the carbon black-filled composites decreased compared to the blank sample due to the plasticizing effect of amino-terminated poly(propylene oxide).

#### 3.1.3. Effect of Amino-Terminated Poly(propylene oxide) on the Mechanical Properties of Carbon Black/Natural Rubber Composites

**Figure 3:**
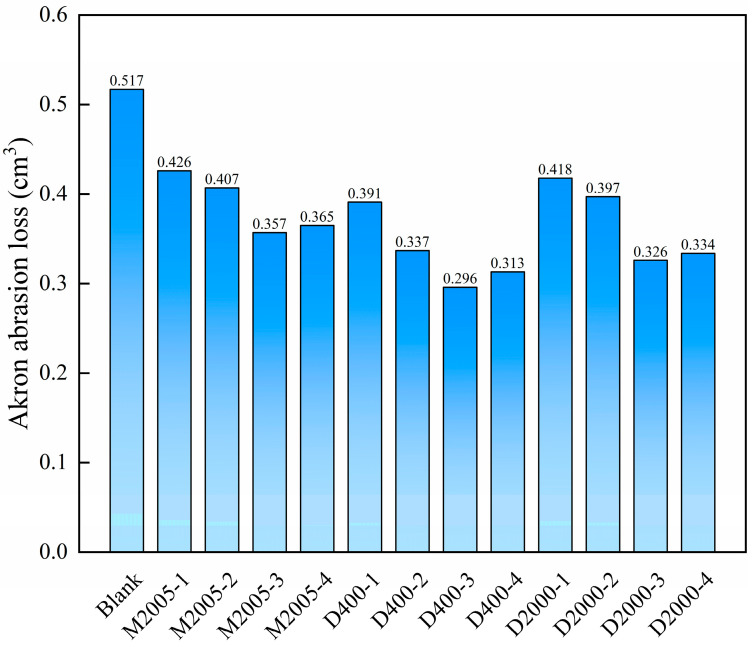
Akron abrasion of carbon black/natural rubber composites with different types and contents of amino-terminated poly(propylene oxide).

Tensile tests were conducted at room temperature using a universal testing machine on carbon black/natural rubber composites containing 1 phr to 4 phr of the three types of amino-terminated poly(propylene oxide). The specific test data are summarized in Table 3. As the content of amino-terminated poly(propylene oxide) increased from 0 to 3 phr, the tensile strength, 100% modulus, and 300% modulus of the composites exhibited an increasing trend, while the elongation at break showed a decreasing trend. These changes can be attributed to the enhanced interfacial interaction between carbon black particles and natural rubber chains facilitated by the amino-terminated poly(propylene oxide). During the tensile deformation of the composite material, the rubber chains experience greater resistance when sliding over the surface of carbon black particles, leading to an increase in both strength and modulus while reducing the elongation at break [28,29]. However, when the content of amino-terminated poly(propylene oxide) increases to 4 phr, both strength and modulus slightly decrease, which may be attributed to the excessive amount of amino-terminated poly(propylene oxide) exhibiting a plasticizing effect. Similarly, M-2005 and D-2000, due to their larger molecular size, exhibit a strong plasticizing effect, resulting in a lower hardness of the composite material compared to the control sample. Among the three types of amino-terminated poly(propylene oxide), the D400-based composite exhibits higher strength and modulus than the M2005-based and D2000-based composites. This is because the di-amino-terminated D400 provides more binding sites with carbon black, leading to stronger interfacial interactions between carbon black and natural rubber in the D400 composite. In contrast, although D2000 is also di-amino-terminated, its larger molecular weight results in partial compatibility with natural rubber, preventing some D2000 from interacting with carbon black. The wear resistance of the composite material can be evaluated using Akron abrasion testing. The samples were tested at room temperature using an Akron abrasion tester, and the Akron abrasion loss of carbon black/natural rubber composites with different types and contents of amino-terminated poly(propylene oxide) is shown in Figure 3. As the content of the three types of amino-terminated poly(propylene oxide) increased from 1 phr to 3 phr, the wear resistance of the carbon black/natural rubber composites improved, likely due to the enhanced dispersion of carbon black within the composite. However, when the amino-terminated poly(propylene oxide) content reached 4 phr, the wear resistance of the composite deteriorated. This trend is consistent with the variations in tensile strength and modulus, as these mechanical properties influence the wear resistance of rubber composites [30].

**Figure 4:**
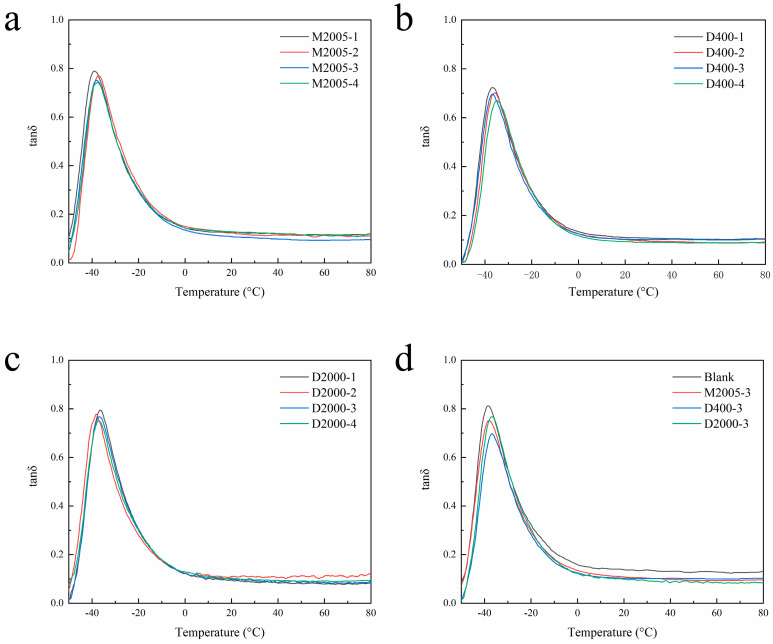
(**a**–**c**) Tan δ–temperature curves of composites containing 1–4 phr of M2005, D400, and D2000. (**d**) Tan δ–temperature curves of composites with 3 phr of the three types of amino-terminated poly(propylene oxide).

Dynamic mechanical analysis (DMA) was conducted on composites with M2005, D400, and D2000 contents ranging from 1 phr to 4 phr, and the temperature-dependent tan δ curves were obtained, as shown in Figure 4. From Figure 4a–c, it can be observed that the tan δ peak values at T_g_ for all three composites decrease with increasing amino-terminated poly(propylene oxide) content. This phenomenon may be attributed to the ability of amino-terminated poly(propylene oxide) to enhance the interfacial interaction between carbon black and natural rubber. The carbon black network captures more free rubber chains, thereby reducing the proportion of natural rubber available for glass transition, which leads to a decrease in the tan δ peak value at T_g_ in the composites [31]. From Figure 4d, it can be observed that the tan δ peak value at T_g_ is the lowest for the D400 composite, followed by the D2000 composite, and the highest for the M2005 composite. This may be because D400 has one more amino group than M2005, resulting in stronger interfacial interactions between carbon black and natural rubber in the D400 composite. In contrast, due to its higher molecular weight, a portion of D2000 dissolves in natural rubber, leading to weaker interfacial interactions between carbon black and natural rubber in the D2000 composite compared to the D400 composite. Rolling resistance is a critical parameter for carbon black/natural rubber composites used as tire treads, as it reflects the energy loss caused by the conversion of mechanical energy into heat due to the hysteresis of the rubber composite [32,33]. Typically, the tan δ value of the rubber composite measured at 60 °C under a dynamic strain of 5–7% and a frequency of 10 Hz is used as a predictive indicator of rolling resistance [34]. As shown in Figure 4d, the tan δ values at 60 °C for the three composites follow the order: D2000 < D400 < M2005 < Blank. In the composites containing amino-terminated poly(propylene oxide), the dispersion of carbon black was improved, which weakened the formation of carbon black aggregates and networks to some extent. This reduction in CB particle agglomeration led to decreased internal friction between CB particles, resulting in lower rolling resistance compared to the blank sample [35]. The D2000 composite exhibited an even lower tan δ value at 60 °C, possibly due to the higher molecular weight of D2000, which provided a stronger plasticizing effect and further reduced internal friction between CB particles.

#### 3.1.4. Effect of Amino-Terminated Poly(propylene oxide) on the Aging Resistance of Carbon Black/Natural Rubber Composites

In order to investigate the effect of amino-terminated poly(propylene oxide) on the aging resistance of carbon black/natural rubber composites, we aged the blank samples and D400-3 composites by thermo-oxidative aging in an oven at 70 °C for 3 days, and then removed and placed for a certain period of time to determine the mechanical properties of the aged composites. The tensile strength of the blank sample after aging for 3 days was 20.56 MPa, which was 10.9% lower than that before aging, and its elongation at break was 428.27%, which was 18.6% lower than that before aging. The tensile strength of the D400-3 composite after aging was 23.87 MPa, which was 10.9% lower than that before aging, and its elongation at break was 382.74%, which was 15.2% lower than that before aging. It can be found that amino-terminated poly(propylene oxide) has little effect on the aging resistance of natural rubber composites.

### 3.2. Effect of Different Silica-to-Carbon Black Ratios on the Hybrid Filler/Natural Rubber Composites

After optimizing the type and dosage of amino-terminated poly(propylene oxide) in the pure carbon black system, we proceeded to enhance the resistivity of natural rubber to make it suitable for electrical spark testing. To achieve this, we prepared natural rubber composites with four different hybrid filler ratios of carbon black to silica: 40:10, 30:20, 20:30, and 10:40. D-400 was incorporated into the system at a fixed mass ratio with carbon black as a dispersing agent for both carbon black and silica. The effect of different carbon black-to-silica ratios on CB/SiO_2_ hybrid filler/natural rubber composites was investigated.

#### 3.2.1. Dispersion of Fillers in Natural Rubber Composites with Different Silica-to-Carbon Black Ratios

**Figure 5:**
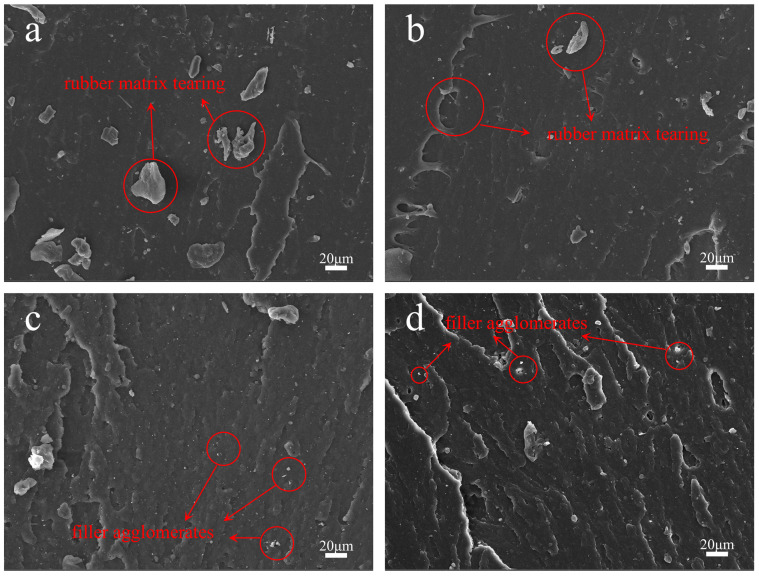
(**a**–**d**) SEM images of the composites with CB:SiO_2_ ratios of 40:10, 30:20, 20:30, and 10:40.

We prepared natural rubber composites with different carbon black and silica ratios and observed the tensile fracture cross-sections using SEM. The obtained images are shown in Figure 5. It can be seen that the agglomerates of both silica and carbon black are minimal in the four mixed-filler composites, indicating good dispersion of both fillers. Since amino-terminated poly(propylene oxide) can interact with the surface groups of both silica and carbon black, enhancing their compatibility with natural rubber, and Si-69 can also assist in improving the dispersion of silica; the fillers in composites with high silica content still exhibit good dispersion.

#### 3.2.2. Mechanical Properties of Natural Rubber Composites with Different Silica-to-Carbon Black Ratios

**Figure 6:**
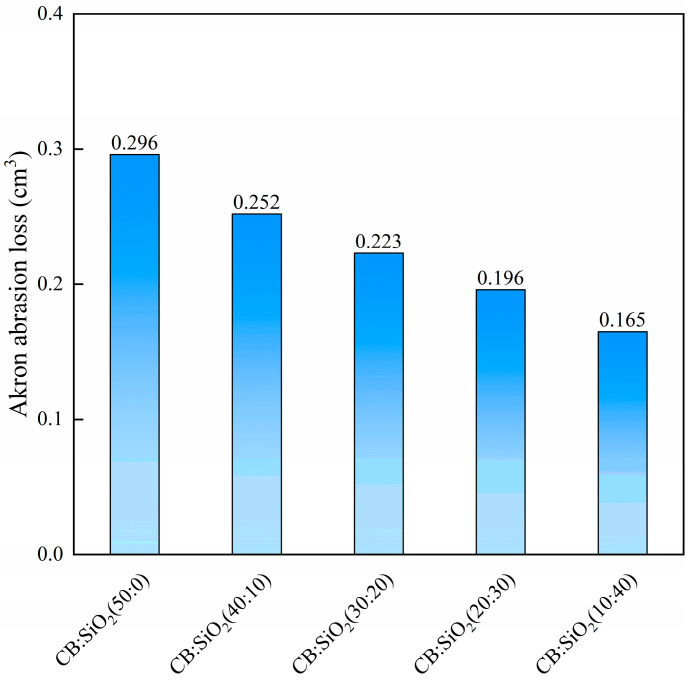
Akron abrasion of hybrid filler/natural rubber composites with different silica-to-carbon black ratios.

We prepared natural rubber composites with different carbon black-to-silica ratios incorporating D400 and measured their stress–strain curves. The detailed mechanical properties data are summarized in Table 4. As the silica content in the hybrid filler system increased, the composite’s strength, modulus, and hardness gradually decreased, while the elongation at break progressively increased. This can be attributed to the higher cohesive energy of silica particles, which makes them more prone to agglomeration in the natural rubber matrix. Consequently, silica exhibits lower reinforcing efficiency compared to carbon black; however, its contribution to elongation at break is greater than that of carbon black [16,36]. Under the influence of the dispersing agent, the strength and modulus of hybrid filler/natural rubber composites with silica content at or below 30 phr are superior to those of the control sample. The Akron abrasion resistance of hybrid filler/natural rubber composites with different silica-to-carbon black ratios is shown in Figure 6. As the silica content increases, the wear resistance of the composites improves, which is contrary to the trend observed in strength and modulus. Through multiple observations during the testing process, we found that when the grinding wheel interacts with the hybrid filler samples, the increased resilience of the hybrid filler/natural rubber composites may cause the grinding wheel to be pushed away, thereby reducing the effective abrasion time and leading to lower Akron abrasion loss.

In the work of Zou, M et al. [9], epoxy natural rubber composites were prepared as tire tread materials using a hybrid filler system of 40 phr carbon black and 10 phr silica, achieving a tensile strength of 24.50 MPa and a 300% modulus of 15.10 MPa. In comparison, the CB:SiO_2_(40:10) composite in this study exhibited a tensile strength of 26.37 MPa and a 300% modulus of 17.07 MPa. Anand, G et al. [37] investigated the hybrid reinforcement of carbon black/silica in natural rubber/styrene–butadiene rubber (NR/SBR) blends. Their rubber composite filled with 30 phr carbon black and 20 phr silica exhibited a tensile strength of 17 MPa and a 100% modulus of 3.2 MPa. In comparison, the CB:SiO_2_(30:20) composite in this study achieved a tensile strength of 24.03 MPa and a 100% modulus of 3.01 MPa. This indicates that the carbon black/silica hybrid-filled rubber composites developed in this study can meet general industrial requirements in terms of mechanical performance.

**Figure 7:**
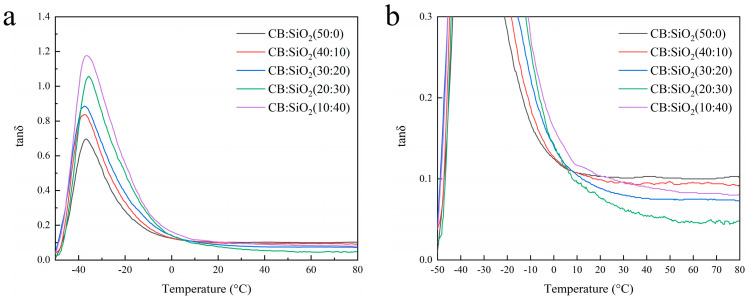
(**a**) Tan δ–temperature curves of hybrid filler/natural rubber composites with different silica-to-carbon black ratios. (**b**) Magnified view of (**a**).

The vulcanized hybrid filler/rubber composites were tested using a dynamic mechanical analyzer (DMA), and the tan δ–temperature curves of the hybrid filler/natural rubber composites with different silica-to-carbon black ratios were obtained, as shown in Figure 7. From Figure 7a, it can be observed that as the silica content increases, the tan δ peak value at T_g_ also increases. This phenomenon may be attributed to the weaker interfacial interaction between silica and natural rubber compared to that of carbon black and natural rubber. As a result, a higher proportion of natural rubber chains remains uncaptured by the filler network, allowing more rubber to undergo the glass transition, thereby leading to an increase in the tan δ peak at T_g_. The advantage of silica-filled natural rubber composites lies in their low rolling resistance and high wet skid resistance [38,39], which can be observed in Figure 7b. The tan δ of the hybrid filler composites at 0 °C is higher than that of the pure carbon black composite, while at 60 °C, the tan δ is lower than that of the pure carbon black composite. The composite with a silica-to-carbon black ratio of 40:10 exhibits the best wet skid resistance, whereas the composite with a silica-to-carbon black ratio of 30:20 demonstrates the lowest rolling resistance.

### 3.3. Electrical Resistivity of Natural Rubber Composites with Different Filler Compositions

The volume resistivity and surface resistivity of natural rubber composites with different filler compositions were measured at room temperature. The results are shown in Table 5. When the carbon black content is high, the conductive particles are either in direct contact or separated by very small gaps, facilitating the formation of conductive pathways. As a result, the volume resistivity and surface resistivity of the composites are relatively low [40]. At this stage, as the silica content increases, only a small portion of the conductive pathways formed by interconnected carbon black particles are disrupted, resulting in a relatively small increase in resistivity. When the silica content reaches a critical threshold (20 phr), most of the conductive pathways are on the verge of disconnection, and a slight increase in silica leads to a resistivity increase by several orders of magnitude. This phenomenon is known as the “percolation phenomenon” and the corresponding critical threshold is referred to as the “percolation threshold” [41,42]. Beyond this point, further increasing the silica content causes the conductive carbon black particles to become relatively isolated within the rubber matrix, preventing the formation of a conductive network. Consequently, the increase in resistivity is less significant, and the composite exhibits overall insulating behavior. The resistivity of the CB:SiO_2_(40:10) composite is the same as that of the CB40 composite, both at 10^6^ Ω·cm. However, the resistivity of the CB:SiO_2_(30:20) composite is two orders of magnitude higher than that of the CB30 composite, while the resistivity of the CB:SiO_2_(20:30) composite is four orders of magnitude higher than that of the CB20 composite. This is because the amount of silica at low silica content is insufficient to disrupt the conductive pathways of carbon black. As the silica content increases, its insulating effect gradually becomes more pronounced.

Next, we conducted spark testing on natural rubber composites with different filler compositions using a voltage of 6000 V (3000 V/mm). During testing, composites with low resistivity that were unsuitable for spark testing immediately generated sparks due to self-discharge. In contrast, composites suitable for spark testing only produced sparks when the air within defects was ionized and broken down. After multiple tests, the CB20 composite was found to be inconsistent in its suitability for spark testing, exhibiting instability. However, the CB:SiO_2_(30:20) composite was confirmed to be suitable for spark testing. By incorporating silica into the composite system, we successfully increased the carbon black loading in spark-testable composites by 10 phr, which significantly enhanced the mechanical properties of the rubber composites.

## 4. Conclusions

We utilized different types of amino-terminated poly(propylene oxide) as interfacial dispersants for silica/carbon black hybrid-filled natural rubber composites and developed a low-conductivity natural rubber composite suitable for spark testing while meeting general industrial mechanical performance requirements. In the pure carbon black/natural rubber composite system, we found that three types of amino-terminated poly(propylene oxide) effectively improved the dispersion of carbon black, enhanced the mechanical strength of the composite—particularly the 300% modulus—but also accelerated the vulcanization rate. In the hybrid filler system, both carbon black and silica exhibited good dispersion. The natural rubber composite with a filler composition of 30 phr carbon black and 20 phr silica demonstrated a tensile strength of 24.03 MPa, a 300% modulus of 15.16 MPa, and an elongation at break of 455.15%. Additionally, this composite exhibited a volume resistivity of 5.52 × 10^9^ Ω·cm and a surface resistivity of 3.90 × 10^9^ Ω, making it suitable for spark testing in defect detection.

## Figures and Tables

**Table 1:** Formulation of rubber composites ^c^.

Materials (phr ^a^)	NR	N220	SiO_2_	M-2005	D-400	D-2000	Si-69 ^b^
Blank	100	50					
M2005-1	100	50		1			
M2005-2	100	50		2			
M2005-3	100	50		3			
M2005-4	100	50		4			
D400-1	100	50			1		
D400-2	100	50			2		
D400-3	100	50			3		
D400-4	100	50			4		
D2000-1	100	50				1	
D2000-2	100	50				2	
D2000-3	100	50				3	
D2000-4	100	50				4	
CB:SiO_2_(40:10)	100	40	10		2.4		1
CB:SiO_2_(30:20)	100	30	20		1.8		2
CB:SiO_2_(20:30)	100	20	30		1.2		3
CB:SiO_2_(10:40)	100	10	40		0.6		4

^a^ Parts per hundreds of rubber (phr). ^b^ The optimal content of Si-69 was determined to be 10% of the silica mass in previous studies. ^c^ The remaining components and their dosages are as follows: SA 2 phr; ZnO 4 phr; RD 1 phr; 4010 NA 1.5 phr; T50 7 phr; NS 1.5 phr; S 1.5 phr.

**Table 2:** Vulcanization data of carbon black/rubber composites with different types and contents of amino-terminated poly(propylene oxide).

Sample	T_10_/min:s	T_90_/min:s	M_L_/d N·m	M_H_/d N·m
Blank	5:45	13:40	1.32	19.24
M2005-1	4:53	12:18	1.35	19.03
M2005-2	4:50	10:30	1.44	18.29
M2005-3	4:19	9:29	1.40	17.60
M2005-4	4:00	9:28	1.44	17.59
D400-1	2:29	8:06	1.40	18.44
D400-2	1:21	4:18	1.53	18.10
D400-3	0:51	3:25	1.57	18.06
D400-4	0:40	3:12	1.57	18.13
D2000-1	5:18	11:35	1.38	15.83
D2000-2	4:14	9:19	1.30	17.59
D2000-3	3:28	7:58	1.31	16.67
D2000-4	2:47	6:41	1.40	18.53

**Table 3:** Mechanical properties of carbon black/natural rubber composites with different types and contents of amino-terminated poly(propylene oxide).

Sample	Tensile Strength/MPa	Elongation at Break/%	M100%/MPa	M300%/MPa	Hardness (Shore A)
Blank	23.07 ± 0.28	526.35 ± 21.12	1.96 ± 0.03	10.38 ± 0.21	66
M2005-1	24.58 ± 0.23	521.35 ± 14.99	2.31 ± 0.11	11.94 ± 0.17	62
M2005-2	25.70 ± 0.47	516.70 ± 15.94	3.00 ± 0.04	14.11 ± 0.34	61
M2005-3	26.19 ± 0.38	508.40 ± 29.86	3.00 ± 0.06	14.40 ± 0.48	61
M2005-4	25.40 ± 0.27	500.00 ± 11.66	2.97 ± 0.07	14.13 ± 0.26	60
D400-1	24.58 ± 0.16	494.30 ± 20.40	2.57 ± 0.04	13.28 ± 0.42	66
D400-2	26.85 ± 0.34	470.60 ± 7.44	3.36 ± 0.06	16.35 ± 0.37	66
D400-3	27.26 ± 0.12	451.55 ± 18.05	4.39 ± 0.08	18.12 ± 0.35	67
D400-4	26.72 ± 0.52	445.45 ± 26.87	3.78 ± 0.12	17.30 ± 0.67	65
D2000-1	23.74 ± 0.24	480.70 ± 18.95	2.65 ± 0.03	13.56 ± 0.30	65
D2000-2	24.28 ± 0.38	463.00 ± 8.94	2.99 ± 0.05	14.68 ± 0.83	64
D2000-3	25.20 ± 0.47	467.70 ± 12.36	3.17 ± 0.06	15.14 ± 0.14	65
D2000-4	24.20 ± 0.19	437.55 ± 27.45	3.20 ± 0.04	15.39 ± 0.27	65

**Table 4:** Mechanical properties of natural rubber composites with different silica-to-carbon black ratios.

Filler Composition	D400 Content/phr	Tensile Strength/MPa	Elongation at Break/%	M100%/MPa	M300%/MPa	Hardness (Shore A)
CB:SiO_2_(50:0)	3.0	27.26 ± 0.12	451.55 ± 18.05	4.39 ± 0.08	18.12 ± 0.35	67
CB:SiO_2_(40:10)	2.4	26.37 ± 0.78	438.85 ± 26.34	3.10 ± 0.04	17.07 ± 0.43	65
CB:SiO_2_(30:20)	1.8	24.03 ± 0.34	455.15 ± 29.62	3.01 ± 0.02	15.16 ± 0.15	62
CB:SiO_2_(20:30)	1.2	23.23 ± 0.62	481.70 ± 16.71	2.18 ± 0.06	12.81 ± 0.28	58
CB:SiO_2_(10:40)	0.6	22.82 ± 0.15	542.40 ± 27.89	1.62 ± 0.07	10.07 ± 0.39	55

**Table 5:** Volume resistivity, surface resistivity, and spark testing results of composites with different filler compositions.

Filler Composition	Volume Resistivity/Ω·cm	Surface Resistivity/Ω	Spark Testing
CB50	4.13 × 10^6^	3.60 × 10^6^	Not applicable
CB40	8.30 × 10^6^	2.42 × 10^6^	Not applicable
CB30	3.58 × 10^7^	4.89 × 10^7^	Not applicable
CB20	1.47 × 10^9^	9.83 × 10^8^	Unstable
CB10	8.93 × 10^9^	3.27 × 10^9^	Applicable
CB:SiO_2_(40:10)	8.75 × 10^6^	9.30 × 10^6^	Not applicable
CB:SiO_2_(30:20)	5.52 × 10^9^	3.90 × 10^9^	Applicable
CB:SiO_2_(20:30)	6.14 × 10^13^	2.57 × 10^13^	Applicable
CB:SiO_2_(10:40)	1.01 × 10^14^	3.84 × 10^14^	Applicable

## Data Availability

Data are contained within the article.
